# Myeloid-Derived Suppressor Cells in Hematologic Diseases: Promising Biomarkers and Treatment Targets

**DOI:** 10.1097/HS9.0000000000000168

**Published:** 2019-01-28

**Authors:** Nikoleta Bizymi, Sunčica Bjelica, Astrid Olsnes Kittang, Slavko Mojsilovic, Maria Velegraki, Charalampos Pontikoglou, Mikael Roussel, Elisabeth Ersvær, Juan Francisco Santibañez, Marie Lipoldová, Helen A. Papadaki

**Affiliations:** 1 Hemopoiesis Research Laboratory, School of Medicine, University of Crete and Department of Hematology, University Hospital of Heraklion, Heraklion, Greece; 2 Graduate Program Molecular Basis of Human Disease, School of Medicine, University of Crete, Heraklion, Greece; 3 Department of Molecular Oncology, Institute for Medical Research, University of Belgrade, Belgrade, Republic of Serbia; 4 Department of Clinical Science, University of Bergen, Bergen, Norway; 5 Division of Hematology, Department of Medicine, Haukeland University Hospital, Bergen, Norway; 6 Laboratory of Experimental Hematology and Stem Cells, Institute for Medical Research, University of Belgrade, Belgrade, Serbia; 7 Department of Immunology and Microbiology, Hollings Cancer Center, Medical University of South Carolina, Charleston, SC, USA; 8 CHU de Rennes, Pole de Biologie, Rennes, France; 9 INSERM, UMR U1236, Université Rennes 1, EFS Bretagne, Equipe Labellisée Ligue Contre le Cancer, Rennes, France; 10 Laboratoire d’Hématologie, CHU Pontchaillou, Rennes Cedex, France; 11 Department of Biomedical Laboratory Scientist Education, Western Norway University of Applied Sciences, Bergen, Norway; 12 Centro Integrativo de Biología y Química Aplicada (CIBQA), Universidad Bernardo O’Higgins, Santiago, Chile; 13 Laboratory of Molecular and Cellular Immunology, Institute of Molecular Genetics AS CR, Prague, Czech Republic

## Abstract

Myeloid-derived suppressor cells (MDSC) are a heterogeneous group of immature myeloid cells that exist at very low numbers in healthy subjects but can expand significantly in malignant, infectious, and chronic inflammatory diseases. These cells are characterized as early-MDSCs, monocytic-MDSCs, and polymorphonuclear-MDSCs and can be studied on the basis of their immunophenotypic characteristics and their functional properties to suppress T-cell activation and proliferation. MDSCs have emerged as important contributors to tumor expansion and chronic inflammation progression by inducing immunosuppressive mechanisms, angiogenesis and drug resistance. Most experimental and clinical studies concerning MDSCs have been mainly focused on solid tumors. In recent years, however, the implication of MDSCs in the immune dysregulation associated with hematologic malignancies, immune-mediated cytopenias and allogeneic hemopoietic stem cell transplantation has been documented and the potential role of these cells as biomarkers and therapeutic targets has started to attract a particular interest in hematology. The elucidation of the molecular and signaling pathways associated with the generation, expansion and function of MDSCs in malignant and immune-mediated hematologic diseases and the clarification of mechanisms related to the circulation and the crosstalk of MDSCs with malignant cells and other components of the immune system are anticipated to lead to novel therapeutic strategies. This review summarizes all available evidence on the implication of MDSCs in hematologic diseases highlighting the challenges and perspectives arising from this novel field of research.

## Introduction

References to cells of myeloid origin that promote tumor progression through immune evasion mechanisms while also induce inflammatory and hemopoietic responses, go back to the 1970s.^1^ These myeloid cells display immunosuppressive properties and expand particularly in neoplastic, infectious, and inflammatory diseases; they were initially characterized as *natural suppressor* or *veto* or *null cells* because of the absence of surface markers of T-cells, B-cells, natural killer (NK) cells or macrophages and later as *immature myeloid cells* or *myeloid suppressor cells* to denote their main biologic properties.^1^ In 2007, the term myeloid derived suppressor cells (MDSCs) was introduced as the best to reflect the origin and functional trait of these cells despite the heterogeneity in their phenotypic, genomic and biochemical characteristics.^2^ In recent years, MDSCs have been recognized as important immune regulators, potential biomarkers and even therapeutic targets in cancer and other diseases associated with chronic inflammation including infectious diseases, autoimmune diseases and trauma, among others.^3,4^

In humans, MDSCs are identified as CD11b^+^CD33^+^HLA-DR^−/low^ cells.^5^ They can be further divided into 2 distinct populations with the main difference being the expression of CD14 (monocytic—MDSCs, M-MDSCs) or CD15 (polymorphonuclear—MDSCs, PMN-MDSCs) surface molecules. M-MDSCs are morphologically identical to conventional monocytes from which they can be distinguished on the basis of HLA-DR expression. PMN-MDSCs can be distinguished from conventional PMN based on their low-density properties following centrifugation over density gradient as well as on the expression of the lectin type oxidized LDL receptor 1 (LOX-1).^3,6^ A third, minor population of MDSCs has been recognized, the early-stage MDSCs (e-MDSCs), which express neither CD15 nor CD14; these cells are characterized as Lin^−^ (CD3, CD14, CD15, CD19, CD56)HLA-DR^−^CD33^+^ and comprise immature progenitor and precursor cells with myeloid colony-forming activity.^5^ In mice, MDSCs are characterized by the expression of Gr1 and CD11b and can also be divided into PMN-MDSCs (CD11b^+^Ly6G^+^Ly6C^low^ cells), M-MDSCs (CD11b^+^Ly6G^−^Ly6C^high^), and non-PMN-MDSCs/non-M-MDSCs (CD11b+Ly6G^med^Ly6C^med^ cells).^5,7^ Notably, the term granulocytic-MDSCs (G-MDSCs) has previously been used for the definition of PMN-MDSCs in both human and mice.

**Figure 1:**
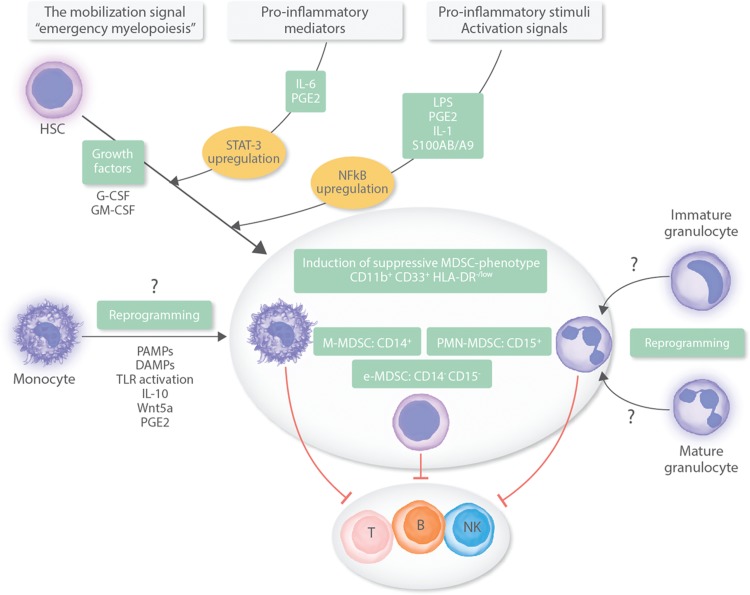
**Proposed signals for MDSC generation.** In humans, MDSCs are identified as CD11b^+^CD33^+^HLA-DR^−/low^ cells and are classified by the expression of CD14 as monocytic-MDSCs (M-MDSCs) or CD15 as polymorphonuclear-MDSCs (PMN-MDSCs). A minor population of MDSCs, the early stage MDSCs (e-MDSCs), expresses neither CD15 nor CD14. The fundamental functional characteristic of MDSCs is the capacity to suppress immune cells, predominantly T-cells and to a lesser degree B-cells and NK-cells. MDSCs arise under inflammatory conditions due to an increased demand for myeloid cells (emergency myelopoiesis); they expand from the hematopoietic stem cell (HSC) as immature cells in the bone marrow (BM) or extramedullary, and migrate into the peripheral blood (PB) where their terminal differentiation is blocked transforming into functionally active MDSCs. Two types of signals are required for MDSCs’ generation: the expansion/mobilization signal through growth factors such as granulocyte and granulocyte/monocyte colony stimulating factors (G-CSF and GM-CSF, respectively) and proinflammatory mediators such as interleukin-6 (IL-6) and prostaglandin E2 (PGE2) resulting in upregulation of STAT3 in myeloid progenitor cells; and the activation signal mediated through proinflammatory stimuli such as lipopolysaccharides (LPS), PGE2, IL-1, and S100A8/A9 resulting in NF-κB upregulation and induction of the suppressive MDSC phenotype. M-MDSCs may also arise by reprogramming of monocytes through pathogen- or danger-associated molecular patterns (PAMPs or DAMPs, respectively) and Toll-like receptor (TLR) activation as well as through certain cytokines and mediators such as IL-10, Wnt5a, and PGE2. PMN-MDSCs may also represent an activation stage of PMNs derived from immature or mature granulocytes.

The precise mechanisms underlying the generation of MDSCs remain largely unknown. MDSCs are likely to arise under inflammatory conditions when there is an increased demand for myeloid cells (emergency myelopoiesis); they then expand as immature cells in the bone marrow (BM) or even extramedullary (mainly in the spleen) and migrate into the peripheral blood (PB) where their terminal differentiation is blocked finally transforming into functionally active MDSCs. According to this model, 2 signals are required for MDSCs’ generation; the expansion/mobilization signal mediated mainly through growth factors such as granulocyte and granulocyte/monocyte colony stimulating factors (G-CSF and GM-CSF, respectively) and proinflammatory mediators such as interleukin-6 (IL-6) and prostaglandin E2 (PGE2) resulting in upregulation of the signal transducer and activator of transcription (STAT)-3 in myeloid progenitor cells; and the activation signal mediated through proinflammatory stimuli such as lipopolysaccharides (LPS), PGE2, IL-1 and S100A8/A9 resulting in NF-κB upregulation and induction of the suppressive MDSC phenotype. Recent evidence suggests that M-MDSCs may also arise by reprogramming of monocytes through pathogen- or danger-associated molecular patterns (PAMPs or DAMPs, respectively) and Toll-like receptor (TLR) activation as well as through certain cytokines and mediators such as IL-10, Wnt5a, and PGE2.^8^ Another hypothesis, although still controversial, indicates that PMN-MDSCs may represent an activation stage of PMNs derived from immature or mature granulocytes^8^ (Fig. 1).

The fundamental functional characteristic of MDSCs is the capacity to suppress immune cells, predominantly T-cells and to a lesser degree B-cells and NK-cells.^1,3,9^ Main transcription factors involved in the suppressive function of MDSCs include STAT3, hypoxia inducible factor 1a (HIF-1a) and CCAAT/enhancer binding protein b (C/EBPb).^1,3,9^ Effector molecules produced by MDSCs include arginase-1, which induces l-arginine deprivation and causes nitrosylation and downregulation of the CD3z part of the T-cell receptor complex; cyclooxygenase (COX)-2 and indoleamine 2,3-dioxygenase (IDO); inducible nitric oxide synthase (iNOS), which leads to the production of NO, induction of T-cell apoptosis and suppression of T-cell proliferation; NADPH oxidase 2 (NOX2), which inhibits the proliferation of T-cells through production of reactive oxygen species (ROS) and nitration of CD3z and major histocompatibility complex (MHC)-I; heme oxygenase 1 (HO-1) which also inhibits T-cell proliferation through carbon monoxide (CO) production; mediators reducing cysteine provision to T-cells by antigen presenting cells; membrane-bound transforming growth factor β1 (TGFβ1), which promotes the anergy of NK-cells and the development of regulatory T cells (Treg); IL-10, which leads to Th2 deviation and type 2 polarization of macrophages; and ADAM metallopeptidase domain 17 (*ADAM*17) which cleaves L-selectin (CD62L) from T-cells leading to their homing in lymph nodes and sites of inflammation.^1,3,9^ In addition to their immune-suppressive properties, MDSCs promote tumor progression and metastasis by affecting the remodeling of the tumor microenvironment and tumor angiogenesis via production of vascular endothelial growth factor (VEGF), basic fibroblast growth factor (bFGF), and matrix metalloproteinase-9 (MMP9).^1,3,9^

The immune system displays a prominent role in the pathogenesis, pathophysiology and response to treatment of patients with hematologic malignancies, BM failure syndromes and autoimmune disorders^10,11^; therefore, immune modulating agents (i.e., lenalidomide, monoclonal antibodies, hypomethylating drugs, signal transduction inhibitors among others) have significantly improved the outcome of these patients.^10,11^ As expected, the possible implication of MDSCs in the immune dysregulation associated with these disease entities and their potential role as biomarkers and therapeutic targets has started to attract a particular interest in hematology.^10,11^ This is further triggered by the fact that MDSCs in addition to their immunosuppressive properties on T-cells can also interact with the mesenchymal stromal cells (MSCs) in the BM through shared molecules and mechanisms and this interplay may alter the immunoregulatory properties of the BM microenvironment and consequently, the disease pathophysiology and response to treatment.^12^ This review summarizes all available evidence on the implication of MDSCs in hematologic malignancies and immune-mediated BM failure syndromes and cytopenias highlighting the challenges and perspectives arising from this novel field of research.

## Myeloproliferative neoplasms and acute leukemia

Myeloproliferative neoplasms (MPNs) are a group of hemopoietic stem cell disorders characterized by clonal proliferation of myeloid-lineage cells and chronic inflammation.^13^ Classic MPNs include chronic myeloid leukemia (CML), polycythemia vera (PV), essential thrombocythemia (ET), and primary myelofibrosis (PMF). Studies on MDSCs in CML have demonstrated increased frequency of PMN-MDSC subsets in Sokal high-risk patients, expressing high levels of programmed death receptor ligand-1/programmed death receptor-1 (PD-L1/PD-1) and arginase-1.^14,15^ Increased PMN-MDSC and M-MDSC subsets in CML patients at diagnosis have been shown to return to normal levels after treatment with the tyrosine kinase inhibitor (TKI) imatinib and M-MDSCs frequency has been proposed as a prognostic factor in CML patients receiving the TKI dasatinib.^16^ Overall, CML patients appropriately responding to TKI therapies (imatinib, nilotinib, dasatinib), parallel to the reduction of abl-bcr transcripts display a decrease in M-MDSC frequency and immunosuppressive activity and restoration of T-cell and NK-cell immune functions.^17^ Recent evidence also suggests that MDSCs may have a critical role in CML malignant cell immune escape. In particular, it has been shown that the malignant cell expansion in CML is maintained by a small subset of CD34^+^/CD38^−^ leukemic stem cells that may escape immune cell surveillance within immunosuppressive BM niches consisting of populations of MSCs and PMN-MDSCs with T-cell suppressive capacity.^18^ It is therefore reasonable to hypothesize that targeting of MDSCs in CML may restore the T-cell mediated leukemia surveillance and improve further patients’ long-term outcome.

As regards to the Philadelphia negative MPNs, namely PV, ET, and PMF, a number of studies have documented abnormal reserves and function of immune cells including increase of monocyte/macrophages, altered regulatory T-cell frequency, NK-cell dysfunction, and expansion of MDSCs.^19^ Recent evidence also suggests that although MDSCs are significantly elevated in MPNs, no differences can be identified in their frequency among different MPN categories and no correlations with JAK2 allele burden can be made.^20^ MPN-derived MDSCs have also been shown to display significantly elevated arginase-1 mRNA and T-cell suppressive activity.^20^

There are only a few studies on MDSCs in acute leukemias clearly showing the negative impact of their presence on the disease prognostic characteristics. A recent study has shown variable but significantly higher number of MDSCs, defined as CD14^−^HLA-DR^−^CD33^+^CD11b^+^ cells, in PB of patients with acute myeloid leukemia (AML) compared to patients with acute lymphoblastic leukemia (ALL) and significant correlation of AML-MDSCs with conventional prognostic factors at diagnosis, namely WBC count, CD34 frequency and nucleophosmin (NPM1) and fms-like tyrosine kinase 3 (FLT3) gene mutations. It has also been shown that CD33^+^CD11b^+^HLA-DR^−/low^ MDSCs accumulate in the BM of AML patients and their presence may have an impact on disease prognosis and patients’ clinical course.^21^ Furthermore, MDSCs levels in newly diagnosed AML patients have been reported to correlate with AML subtype, presence of chromosomal abnormalities and gene alterations, extramedullary involvement, and plasma D-dimer levels. After the induction therapy, MDSCs significantly decrease in patients with complete remission but not in patients with partial or no response while on follow-up and MDSCs’ frequency correlate with minimal residual disease (MRD) levels and Wilms 1 (WT-1) gene detection.^21^ Another study has shown expansion of MDSCs from PB mononuclear cells (PBMCs) following contact with AML cell lines or primary AML cells via tumor-derived extracellular vesicles; it has been postulated that the mucin 1 (MUC1) oncoprotein induces increased expression of c-myc in extracellular vesicles that are then taken-up by the myeloid progenitor cells resulting in downstream effects on cell cycle proteins and selective proliferation of MDSCs.^22^ As regards to ALL, it has been shown that pediatric patients with B-cell ALL display increased number of PMN-MDSCs in PB and BM compared to age-matched healthy individuals and PMN-MDSC frequency correlate with prognostic markers such as MRD and CD20^+^ blast cell counts as well as with response to therapy.^23,24^ PMN-MDSCs from patients in remission have been reported to lose their suppressive function corroborating the effect of these cells in mediating immune evasion mechanisms.^23,24^ All these observations and available evidence implicating MDSCs in both AML and ALL progression and outcome highlight the emerging role of these cells as independent biomarkers and promising targets for the development of novel therapeutic strategies.

## Myelodysplastic syndromes

Myelodysplastic syndromes (MDS) are clonal hemopoietic stem cell disorders characterized by PB cytopenias, dysplastic changes in one or more BM cell lineages and increased risk of AML.^25^ Allogeneic hemopoietic stem cell transplantation (Allo-HSCT) with donor T-cell reactivity against the malignant cells is the only curative therapy for MDS. BM microenvironment in low-risk MDS is characterized by persistent inflammation and expansion of autoimmunity-associated T-helper 17 (Th17) cells whereas expansion of MDSCs and Tregs and reduced number of Th17 characterize high-risk MDS.^26,27^ Lin^−^(CD3/CD14/CD16/CD19/CD20/CD56)HLA-DR^−^CD33^+^ MDSCs in high-risk MDS are capable to induce apoptosis of erythroid precursor cells and their expansion is driven, at least in part, by the interaction of S100A9 protein with the CD33 cell surface antigen.^28^ Immune phenotyping with mass cytometry and unsupervised viSNE analysis of 35-markers per cell of thawed MDS and AML BM samples has revealed that one of the strongest marker signals was the expression of S100A9. This protein is seen in multiple cell types including MDSCs and its expression was more common in MDS without excess of blasts. S100A9 mainly characterizes M-MDSCs and together with its binding partner, S100A8, is included in the DAMP molecules displaying intra- and extracellular functions and inflammatory, antiinflammatory and immune regulatory properties.^29,30^ Varying S100A9 expression resulted in different MDSC functions, with more proinflammatory effect in low risk and immunosuppressive effect in high-risk MDS. S100A9 and CD33 ligand/receptor pair interactions recruit components to the immunoreceptor tyrosine-based inhibition motif (ITIM) finally resulting in secretion of suppressive IL-10 and TGFβ1.^28^ Work based on fresh PB and BM samples from MDS patients confirmed the secretion of these 2 cytokines by MDSCs, but interestingly, this secretion was higher in PMN-MDSCs than in M-MDSCs.^31^ In fact, PMN-MDSC subset dominated the MDSC-expansion in high-risk patients. The BM homing chemokine receptors (CXCR4, CX3CR1) were expressed at a higher level on M-MDSCs in high-risk MDS, and there was different expression of CX3CR1 between healthy donors, low-risk MDS and high-risk MDS patients.^31^

The importance of MDSCs in suppression of hemopoiesis in MDS has been demonstrated in 2 genetically manipulated animal models; the S100A9 transgenic mice, displaying BM accumulation of MDSC and progressive cytopenia and the mDia1/miR146-a double knockout mice, developing age-related inflammatory BM microenvironment and anemia.^28,32^ S100A8/A9 activation of MDSC is through the NF-κB signaling pathway; therefore, we may hypothesize that by targeting this pathway we could reduce MDSCs levels.^33^

Drugs widely used for MDS have lately been shown to affect T-cell polarization, which may suggest effects on MDSCs activity. Azacitidine, the drug of choice in high-risk MDS, has been shown to affect T-cell polarization in the Th17/Treg-axis in high-risk MDS and to influence levels of BM CD57^+^ T-cells, CD57^+^ T-cell degranulation and CD34^+^ BM cell directed cytotoxicity.^34,35^ Specific MDSC-targeting in MDS has so far been aiming at CD33-expressing cells.^36^ So far, BI 836858 (Fc-engineered anti-CD33 moAb) for antibody-dependent cell-mediated cytotoxicity by NK-cells is currently tested in MDS patients in an ongoing Phase I/II clinical trial (ClinicalTrials.gov Identifier: NCT02240706). Additionally, a clinical trial with CD16/IL-15/CD33 (161533) tri-specific killer cell engager for the treatment of CD33-expressing myeloid malignancies, including high-risk MDS, is not yet recruiting (NCT03214666). Overall, the implication and therapeutic targeting of MDSCs in MDS is an interesting, open field of research.

## Lymphomas

Different mice models have been used to study MDSC biology in lymphomas. For example, M-MDSC and PMN-MDSCs from EL4 and EG7 lymphoma models display immunosuppressive capacity associated with increased production of NO and nitrotyrosine (M-MDSCs) and ROS (PMN-MDSCs).^37,38^ Furthermore, MDSCs from A20 B-cell lymphoma model, operate as tolerogenic antigen presenting cells capable of antigen uptake and presentation to tumor-specific Tregs.^39^ In humans, the number of circulating MDSCs has been correlated with poor prognosis in diffuse large B-cell lymphoma (DLBCL),^40,41^ indolent lymphoma,^42^ chronic lymphocytic leukemia,^43,44^ and Hodgkin lymphoma (HL).^40,45^ However, only few of the suppressive mechanisms involved in MDSC biology in these disease entities have been elucidated.^46^ In DLBCLs, an increase of immunosuppressive PMN-MDSC numbers expressing arginase-1 has been noticed, however correlation with clinical outcome was not been documented in all studies.^40,41,47^ Similarly, increased numbers of M-MDSCs (CD14^+^HLA-DR^low^) have been detected in PB from DLBCL patients in various studies.^41,42,48,49^ M-MDSCs from DLBCL patients have been found to overexpress genes involved in MDSC biology such as IL4-R, IL6-R, RELB, STAT3, NFKB, CEBPβ, AIM2, TNFR2, and NOX2. Additionally, the T-cell suppressive effect of MDSCs was mediated by a release of IL-10 and S100A12 and an increase in PD-L1 expression.^41^ Increased circulating PMN-MDSC (CD66b^+^CD33^dim^HLA-DR^−^) numbers compared to healthy donors with elevated arginase-1 activity has been observed in a cohort of 31 patients with indolent lymphomas^40^ whereas increased M-MDSC (CD14^+^HLA-DR^low^) numbers have been detected in another cohort of 22 patients with indolent lymphomas.^42^ Increased PMN-MDSC and M-MDSC numbers and arginase-1 activity have been also identified in PB of HL patients.^40,45^ As regards to T-cell lymphomas, even less is known about the potential role of MDSCs. Patients with mycosis fungoides and Sézary syndrome with stage IB and above have been shown to display increased production of ROS by MDSCs compared to patients with stage IA or healthy controls, despite the normal MDSC numbers and this abnormality was reversed following anti-CD25 denileukin diftitox or IFN-α2b treatment.^50^ Collectively, all the above studies have pointed out the possibility of targeting MDSCs in future therapeutic trials with lymphoma patients by controlling their expansion and/or blocking their immunosuppressive functions.

## Multiple myeloma

Multiple myeloma (MM) is a B-cell malignancy characterized by expansion of monoclonal plasma cells preferentially in the BM and the accumulation of monoclonal immunoglobulins in the PB. It is now widely accepted that the BM microenvironment displays a prominent role in pathophysiology of the disease by providing a protective niche to the plasma cells that promotes the immune-escape, drug-resistance, and angiogenesis.^51^ An interactive crosstalk between the malignant cells and the BM microenvironment is also responsible for many clinical characteristics of the disease such as the osteolytic lesions, anemia and immunosuppression.^51^ According to recent studies, MDSCs are involved in the pathogenesis and progression of MM.^52–54^ Experiments in 5T2 and 5T33MM mice models of MM have shown that the malignant plasma cells can induce the generation and survival of both PMN-MDSCs and M-MDSCs that accumulate in the BM in early stages and in PB at later stages, and display T-cell immunosuppressive activity, through production of NOS, arginase-1 and IL-10, which is highest among M-MDSCs.^55,56^ In the DP42 MM mouse model, however, a more prominent role of the BM PMN-MDSC population was demonstrated for the induction of plasma cell growth and chemoresistance.^57^ The malignant cells secrete factors such as IL-6, GM-CSF, VEGF, IL-1b and exosomes leading to the activation of STAT3 and STAT1 pathways, increase expression of Bcl-xL and Mcl-1 proteins and release of NO enhancing finally the BM angiogenesis as well as the survival and suppressive activity of MDSCs.^55,58,59^ These effects can be further potentiated by the MSCs in MM BM microenvironment.^60,61^ Using the 5TGM1 mouse model it has also been shown that tumor-induced MDSCs, in addition to their immunosuppressive effect, can be differentiated into mature and functional osteoclasts contributing therefore to the bone destruction associated with the disease.^62^ The importance of MDSCs in the development of MM was demonstrated in the S1009 knockout transgenic mice which display defective response to cancer and delay in the development of MM following inoculation of MM cells which is reversed following adoptive transfer of MDSCs.^63^ In humans, early studies have shown that MDSCs isolated from the PB of patients with MM display a T-cell inhibitory effect which can be abrogated by drugs inhibiting arginase-1 and iNOS activity.^64^ Similar to mice models, increased number of PMN-MDSCs with immunosuppressive properties has been reported in the BM and PB of patients with MM at diagnosis and relapse compared to healthy subjects.^63,65,66^ The frequency of PMN-MDSCs has been shown to correlate with the disease activity and it is higher in MM patients compared to patients with monoclonal gammopathy of undetermined significance (MGUS) suggesting that these cells can be used as markers of disease activity and progression.^65–67^ Contradictory results have been published so far regarding M-MDSC numbers and function in patients with MM. Increased numbers of M-MDSCs have been reported in BM and PB of newly diagnosed and relapsed MM patients compared to patients in remission or healthy donors suggesting their potential consideration as prognostic predictors of disease activity^68^ whereas other studies have not identified such differences.^63,65–67^ Discrepancies may be due to different flow-cytometric strategies and different quantitative and qualitative characteristics of patient cohorts.

In summary, all available evidence suggests that MDSCs are increased in the patients with MM and participate in the pathophysiology of the disease by inducing the survival and proliferation of malignant plasma cells both directly and indirectly through their immunosuppressive effects. Therefore, MDSCs can become therapeutic targets for MM. It has been shown that the immunomodulatory drug lenalidomide and the proteasome inhibitor bortezomib are able to downregulate molecules produced by MDSCs in MM; however, they cannot abrogate the number or immunosuppressive function of MDSCs.^66^ These observations emphasize the importance of developing novel agents to overcome the immunosuppressive effects of MDSCs in MM patients.

## Immune-mediated cytopenias

Immune thrombocytopenia (ITP) is a disease entity characterized by low platelet count due to antiplatelet autoantibodies, abnormal effector T-cell activation and inappropriate platelet production in the BM.^69^ Recent evidence suggests that MDSCs have a role in the pathophysiology of the disease but contradictory results have published thus far on the frequency and function of MDSCs at diagnosis probably due to different flow-cytometry strategies, that is, whole blood or PBMCs.^70–72^ Circulating MDSCs in ITP patients increase following immunosuppressive treatment with high dose dexamethasone (DXM) and MDSC numbers correlate with platelet recovery suggesting that PB MDSCs could be used as markers of response to therapy.^70–72^ It has also been shown that PB and splenic MDSCs in ITP patients display impaired immunosuppressive function contributing possibly to the pathogenesis of the disease and that DXM treatment improves the immunomodulatory properties of MDSCs including the production of suppressive cytokines and their T-cell suppressive effects.^70^ The effect of DXM on MDSCs was found to correlate with the transcription factor Ets1 both in ITP patients and a murine model of ITP generated following transfer of splenocytes from CD61 knockout mice immunized with CD61^+^ platelets into severe combined immunodeficient mouse recipients.^70^ Interestingly, adoptive cell transfer with MDSCs alleviated thrombocytopenia and resulted in higher survival rate in the ITP murine model.^70^ Treatment with intravenous immunoglobulin (IVIG) has been reported to increase the number of MDSCs in spleen cell cultures from ITP patients indicating that, in addition to blocking the macrophage Fc receptors, IVIG may ameliorate ITP by increasing MDSC populations similar to DXM.^73^ The findings provide novel insights linking MDSCs with the pathogenesis, disease activity and management of ITP that need further investigation in the clinic.

Chronic idiopathic neutropenia (CIN) is another immune-mediated disorder characterized by prolonged, unexplained reduction in the number of PMN associated in the majority of patients with the presence of activated T-lymphocytes with myelosuppressive properties that induce the apoptotic death of the granulocytic progenitor cells.^74^ Preliminary data on the role of MDSCs have shown low frequency of PB PMN-MDSCs and M-MDSCs in CIN patients and this decrease might contribute to the aberrant T-cell activation and sustained chronic inflammation in CIN, a hypothesis that is currently under investigation.^75^

Aplastic anemia is the prototype of T-cell mediated BM failure syndrome and the potential implication of MDSCs in the abnormal T-cell responses associated with the disease remains an open field for research. In a mouse model of acquired aplastic anemia following deletion of the TGFβ-activated kinase-1 gene in hemopoietic cells, the BM failure was significantly progressed following inactivation of TNFα signaling and was associated with increased capacity of macrophages to prime T-helper type I cell development and reduced ability of MDSCs to suppress T-cell proliferation.^76^

## Graft versus host disease

Allo-HSCT represents the only curative treatment for a number of hematologic malignancies. The beneficial effect of the treatment is regularly implicated by the immunological attack of the recipient tissues, an effect known as graft-versus-host disease (GvHD) which is associated with significant morbidity and mortality.^77^ Based on their immunesuppressive properties, there is an increasing interest in exploring the possible implication of MDSCs in the development of GvHD and their potential effect on the treatment and patients’ clinical outcome.^78,79^ Studies have shown that PMN-MDSCs and M-MDSCs are increased in the PB during G-CSF stem-cell mobilization in human donors^80^ and that the MDSC content of the graft correlates inversely with the risk of acute GVHD risk in patients receiving allogeneic, G-CSF mobilized, PB stem cells.^81,82^ Results from a recent study also showed that accumulation of MDSCs in the graft and in PB during engraftment results in successful control of severe acute GVHD and long-term survival without any influence on the risk of disease recurrence after allo-HSCT.^83^ In accordance with the clinical data are results from murine models demonstrating that the adoptive transfer of donor MDSCs in fully MHC-mismatched allo-HSCT recipients can result in successful control of GvHD without compromising the graft versus tumor effects.^84,85^ Although there are no currently clinical trials using MDSC infusions in GvHD patients, the experimental data from animal models and observations from patients undergoing allo-HSCT indicate that MDSCs represent a promising therapeutic tool for the prevention and therapy of GvHD in the clinic.^86^ Certainly, studies evaluating large patient cohorts and long observational periods are required to clarify the beneficial effects of MDSCs in patients receiving allo-HSCT versus potential risks from infections or immunosuppression.

## Discussion

In recent years, there has been an increasing interest in the investigation of the contribution of MDSCs in the pathogenesis/pathophysiology of hematologic diseases. Although different protocols and strategies have been used for MDSC investigation and characterization in hematologic diseases, there is conclusive evidence suggesting that similar to their role in cancer induction and progression, MDSCs have a decisive role in hematologic malignancies by suppressing the immune reactions against the malignant cells through previously described mechanisms^10,11^ (Table 1). But also, decreased number and defective function of MDSCs may have a contributing role in the hematologic diseases other than malignancies such as immune-mediated cytopenias including ITP or CIN by augmenting the T-cell mediated platelet or neutrophil destruction.^70,75^ Apparently, MDSCs represent regulatory components of the immune system with critical role in malignant and immune disorders of hemopoiesis.

**Table 1:**
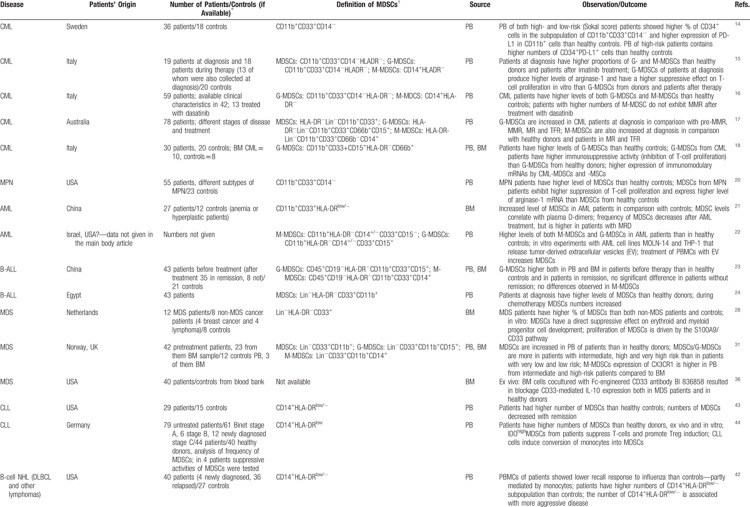
Summary of Representative Studies Investigating the of MDSCs and Their Subpopulations in Different Hematologic Diseases and Their Effects on Disease Outcome

**Table 1 (Continued):**
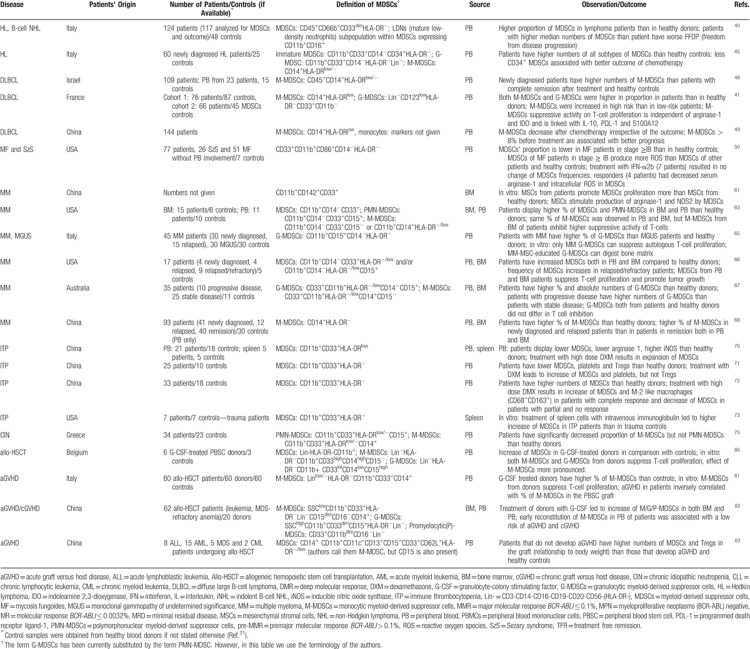
Summary of Representative Studies Investigating the of MDSCs and Their Subpopulations in Different Hematologic Diseases and Their Effects on Disease Outcome

A particular interest for the implication of MDSCs in hematologic diseases originates from the fact that these cells derive from the hemopoietic stem cells which are primarily affected in a number of myeloid malignancies such as MDS, MPN, and acute leukemias. Although MDSCs may be part of the malignant clone, this is not the case in all circumstances^28^; definitely, however, they exert systemic immune suppressive effects and may also contribute to a permissive BM microenvironment where the malignant cells can survive and proliferate by evading host immune-surveillance and antitumor therapies.^11^ In parallel, the malignant cells secrete cytokines, chemokines and growth factors that may sustain and expand MDSCs in a vicious cycle that favors the malignant cell growth and expansion. In lymphoid malignancies, the malignant cells originate from progenitor/precursor or mature cells of the lymphoid system; therefore, any implication of MDSCs in disease pathophysiology, progression and outcome is mediated through their immune-regulatory effects rather than the participation in the malignant population.^39–48^

Apart from the current interest in exploring the reserves, distribution and function of MDSC in hematologic diseases and homogenization of the protocols and strategies for their study, there is also an emerging interest in the potential of developing novel therapeutic strategies targeting MDSCs. Interestingly, a number of therapies currently used for hematologic malignancies have been reported to impact on the number and function of MDSCs and have been extensively reviewed elsewhere.^10,11^ For example, all-trans retinoic acid (ATRA) has been shown to induce the differentiation of MDSCs in mice models and patients with solid tumors resulting in reduction of the number and the immune-suppressive effects of MDSCs^87–90^; its potential effect on MDSCs and consequences in patients with acute promyelocytic leukemia remains to be studied. The pyrimidine nucleoside analog Gemcitabine has been reported to reduce the number of MDSCs inducing therefore an antitumor immunity in mice models and patients with pancreatic cancer.^91,92^ The N-bisphosphonate zoledronic acid has been reported to decrease MDSCs by downregulating MMP9, among other proteins, in patients with pancreatic cancer.^93^ The drug has also been shown to inhibit the bone resorption in MM animal models by reducing the osteoclast formation by MDSCs.^62^ It has also been indicated that the anti-CD38 monoclonal antibody Daratumumab currently used for the eradication of plasma cells in MM patients may additionally eliminate patient MDSCs which also express CD38 according to recently reported data.^94^ Therefore, we may speculate that the well-recognized beneficial effect of all the above treatments in patients with hematologic malignancies might be partially related to their effect on the reserves and functional properties of MDSCs, a hypothesis that needs further investigation. On the other hand, a number of novel therapies currently used in hematologic malignancies target pathways in tumor cells that are also involved in the regulation of physiological processes in MDSCs.^95^ Representative examples are the PI3K and Jak/Stat signaling pathways which are therapeutic targets in lymphomas and MPNs as well as key signaling pathways in MDSCs.^95^ The possible effect of these target therapies on MDSCs and the possibility of an additive effect on the therapeutic outcome is an interesting field of research. It has also been shown that epigenetic modulation of genes such as retinoblastoma 1 (Rb1) may alter the reserves and function of MDSCs.^96^ It is therefore interesting to investigate whether epigenetic regulators including the hypomethylating agents widely used in the treatment of MDS/AML, have also an impact on the MDSC component of the BM microenvironment. Even the FLT3 pathway, representing currently a target for patients with AML and advanced systemic mastocytosis,^97^ has been associated with the expansion and function of MDSCs^98^; therefore the potential alterations of MDSCs in patients treated with the FLT3 inhibitor midostaurin remain to be investigated. Beyond the potential effect of existing therapies on the quantitative and qualitative characteristics of MDSCs, the development of novel therapies targeting MDSC-related signaling pathways or surface molecules, such as CD33, is challenging.

**Figure 2:**
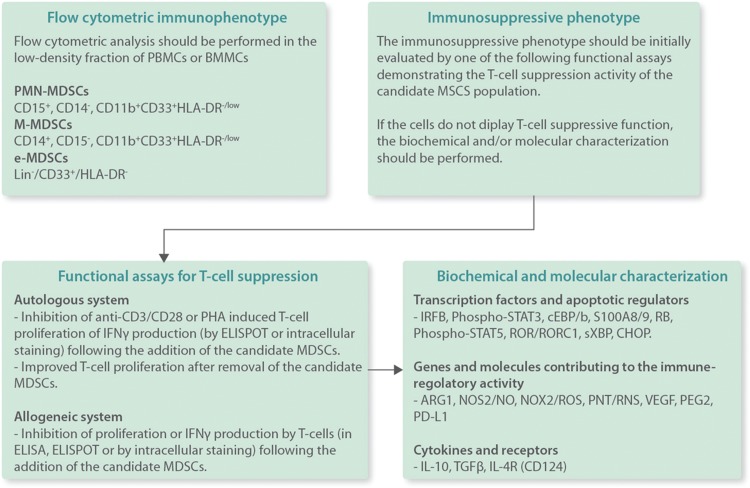
**Algorithm for the characterization of MDSCs in human PB samples or BM aspirates.** For the characterization of a candidate cell population as MDSCs, both the specific immunophenotypic characteristics and the immunosuppressive potential of the cells should be determined. The immunosuppressive potential of the cells should preferentially be performed by a functional assay demonstrating their T-cell suppressive capacity. If the candidate MDSC population lacks a T-cell suppressive function, then this potential should indirectly be demonstrated by the biochemical and/or molecular characterization of the cells. The figure depicts representative functional assays and biochemical and molecular characteristics of MDSCs according to recent recommendations.^5^ ARG1 = arginase-1, BM = bone marrow, BMMCs = bone marrow mononuclear cells, C/EBPb = CCAAT/enhancer binding protein b, CHOP = C/EBP homologous protein, ELISA = enzyme-linked immunosorbent assay, ELISPOT = enzyme-linked immunospot, e-MDSCs = early MDSCs, IFNγ = interferon γ, IL = interleukin, IRF8 = interferon regulatory factor 8, MDSC = myeloid-derived suppressor cells, M-MDSCs = monocytic MDSCs, NOS = nitric oxide synthases, NOX NADPH = oxidase, PB = peripheral blood, PBMCs = peripheral blood mononuclear cells, PD-L1 = programmed death-ligand 1, PGE2 = prostaglandin E2, PHA = phytohemagglutinin, PMN-MDSCs = polymorphonuclear MDSCs, PNT = peroxynitrite, RB = retinoblastoma, RNS = reactive nitrogen species, ROR = RAR-related orphan receptors, ROS = reactive oxygen species, STAT = signal transducer and activator of transcription, sXBP = spliced X-box binding protein, TGF = transforming growth factor, VEGF = vascular endothelial growth factor.

Despite the increasing knowledge on the biology of MDSCs, a number of issues related to the generation, expansion, and circulation of these cells and their precise role in the microenvironment of BM and lymphoid tissues in malignant and immune-mediated hematologic disorders undoubtedly need further investigation. The homogenization of the methods for MDSC assessment is also an important issue because there is considerable variability in how MDSCs have been measured and reported in different studies and the lack of uniform protocols can lead to inconsistencies, uncertainties and erroneous conclusions.^99^ Even the standard method, that is, the immunophenotyping, currently used for the measurement of MDSCs in the low density fraction of PBMCs and BMMCs,^5^ needs further improvement because it prevents the accurate quantification of absolute MDSC numbers whereas markers unique for MDSCs have not been identified so far. Therefore, the functional characterization, and specifically the T-cell suppression activity, remains a key assay for MDSC definition (Fig. 2). Recently, guidelines for the standardization and harmonization of the functional assays were published by a working group from EU COST Mye-Euniter consortium (http://www.mye-euniter.eu) aiming to diminish variations across laboratories for the study of MDSCs from different diseases and tissue sources.^100^

Overall, the better characterization of MDSCs and the elucidation of the molecular and signaling pathways implicated in MDSC generation, expansion and function are anticipated to unravel novel pathogenetic mechanisms in normal and abnormal hemopoiesis and may offer novel therapeutic approaches for patients with malignant and immune-mediated hematologic disorders including patients undergoing allo-ASCT.

## Acknowledgments

The authors would like to thank all members of the COST Action BM1404 Mye-EUNITER for the fruitful discussions during the consortium meetings that helped to the writing of this review. We also gratefully acknowledge the Action for the financial support to NB and SB for short term scientific mission (STSM) exchanges between their Research Laboratories providing expertise that greatly assisted in the writing process of this paper.

## References

[1] Talmadge JE Gabrilovich DI History of myeloid derived suppressor cells (MDSCs) in the macro- and micro-environment of tumour-bearing hosts. *Nat Rev Cancer.* 2013;13:739–752. 24060865 10.1038/nrc3581 PMC4358792

[2] Gabrilovich DI Bronte V Chen SH The terminology issue for myeloid-derived suppressor cells. *Cancer Res.* 2007;67:425–1425. 17210725 10.1158/0008-5472.CAN-06-3037 PMC1941787

[3] Gabrilovich DI Myeloid-derived suppressor cells. *Cancer Immunol Res.* 2017;5:3–8. 28052991 10.1158/2326-6066.CIR-16-0297 PMC5426480

[4] Veglia F Perego M Gabrilovich D Myeloid-derived suppressor cells coming of age. *Nat Immunol.* 2018;19:108–119. 29348500 10.1038/s41590-017-0022-x PMC5854158

[5] Bronte V Brandau S Chen SH Recommendations for myeloid-derived suppressor cell nomenclature and characterization standards. *Nat Commun.* 2016;7:1–10. 10.1038/ncomms12150 PMC4935811 27381735

[6] Condamine T Dominguez GA Youn JI Lectin-type oxidized LDL receptor-1 distinguishes population of human polymorphonuclear myeloid-derived suppressor cells in cancer patients. *Sci Immunol.* 2016;1:aaf8943. 28417112 10.1126/sciimmunol.aaf8943 PMC5391495

[7] Umansky V Adema GJ Baran J Interactions among myeloid regulatory cells in cancer. *Cancer Immunol Immunother.* 2018. 10.1007/s00262-018-2200-6 PMC11028297 30003321

[8] Millrud CR Bergenfelz C Leandersson K On the origin of myeloid-derived suppressor cells. *Oncotarget.* 2017;8:3649–3665. 27690299 10.18632/oncotarget.12278 PMC5356220

[9] Zhao Y Wu T Shao S Phenotype, development, and biological function of myeloid-derived suppressor cells. *Oncoimmunology.* 2016;5:e1004983. 27057424 10.1080/2162402X.2015.1004983 PMC4801459

[10] De Veirman K Van Valckenborgh E Lahmar Q Myeloid-derived suppressor cells as therapeutic target in hematological malignancies. *Front Oncol.* 2014;4:1–11. 25538893 10.3389/fonc.2014.00349 PMC4258607

[11] Younos IH Abe F Talmadge JE Myeloid-derived suppressor cells: their role in the pathophysiology of hematologic malignancies and potential as therapeutic targets. *Leuk Lymphoma.* 2015;56:2251–2263. 25407654 10.3109/10428194.2014.987141

[12] Vladimirovna IL Sosunova E Nikolaev A Mesenchymal stem cells and myeloid derived suppressor cells: common traits in immune regulation. *J Immunol Res.* 2016;2016: 7121580. 10.1155/2016/7121580. PMC4978836 27529074

[13] Barbui T Thiele J Gisslinger H The 2016 WHO classification and diagnostic criteria for myeloproliferative neoplasms: document summary and in-depth discussion. *Blood Cancer J.* 2018;8:15. 29426921 10.1038/s41408-018-0054-y PMC5807384

[14] Christiansson L Söderlund S Svensson E Increased level of myeloid-derived suppressor cells, programmed death receptor ligand 1/programmed death receptor 1, and soluble CD25 in Sokal high risk chronic myeloid leukemia. *PLoS ONE.* 2013;8:1–12. 10.1371/journal.pone.0055818 PMC3561335 23383287

[15] Giallongo C Parrinello N Tibullo D Myeloid derived suppressor cells (MDSCs) are increased and exert immunosuppressive activity together with Polymorphonuclear Leukocytes (PMNs) in chronic myeloid leukemia patients. *PLoS ONE.* 2014;9:1–13. 10.1371/journal.pone.0101848 PMC4094386 25014230

[16] Giallongo C Parrinello NL La Cava P Monocytic myeloid-derived suppressor cells as prognostic factor in chronic myeloid leukaemia patients treated with dasatinib. *J Cell Mol Med.* 2018;22:1070–1080. 29218828 10.1111/jcmm.13326 PMC5783858

[17] Hughes A Clarson J Tang C CML patients with deep molecular responses to TKI have restored immune effectors and decreased PD-1 and immune suppressors. *Blood.* 2017;129:1166–1176. 28049640 10.1182/blood-2016-10-745992

[18] Giallongo C Romano A Parrinello NL Mesenchymal stem cells (MSC) regulate activation of granulocyte-like myeloid derived suppressor cells (G-MDSC) in chronic myeloid leukemia patients. *PLoS ONE.* 2016;11:1–13. 10.1371/journal.pone.0158392 PMC4938578 27391078

[19] Barosi G An immune dysregulation in MPN. *Curr Hematol Malig Rep.* 2014;9:331–339. 25139710 10.1007/s11899-014-0227-0

[20] Wang JC Kundra A Andrei M Myeloid-derived suppressor cells in patients with myeloproliferative neoplasm. *Leuk Res.* 2016;43:39–43. 26943702 10.1016/j.leukres.2016.02.004

[21] Sun H Li Y Zhang Z-F Increase in myeloid-derived suppressor cells (MDSCs) associated with minimal residual disease (MRD) detection in adult acute myeloid leukemia. *Int J Hematol.* 2015;102:579–586. 26358057 10.1007/s12185-015-1865-2

[22] Pyzer AR Stroopinsky D Rajabi H MUC1-mediated induction of myeloid-derived suppressor cells in patients with acute myeloid leukemia. *Blood.* 2017;129:1791–1802. 28126925 10.1182/blood-2016-07-730614 PMC5813734

[23] Liu Y-F Chen Y-Y He Y-Y Expansion and activation of granulocytic, myeloid-derived suppressor cells in childhood precursor B cell acute lymphoblastic leukemia. *J Leukoc Biol.* 2017;102:449–458. 28619949 10.1189/jlb.5MA1116-453RR

[24] Salem ML El-Shanshory MR Abdou SH Chemotherapy alters the increased numbers of myeloid-derived suppressor and regulatory T cells in children with acute lymphoblastic leukemia. *Immunopharmacol Immunotoxicol.* 2018;40:158–167. 29388481 10.1080/08923973.2018.1424897

[25] Arber DA Orazi A Hasserjian R The 2016 revision to the World Health Organization classi fi cation of myeloid neoplasms and acute leukemia. *Blood.* 2016;127:2391–2406.27069254

[26] Kordasti SY Afzali B Lim Z IL-17-producing CD4+ T cells, pro-inflammatory cytokines and apoptosis are increased in low risk myelodysplastic syndrome. *Br J Haematol.* 2009;145:64–72. 19210506 10.1111/j.1365-2141.2009.07593.x

[27] Gabrilovich DI Nagaraj S Myeloid-derived suppressor cells as regulators of the immune system. *Nat Rev Immunol.* 2009;9:162–174. 19197294 10.1038/nri2506 PMC2828349

[28] Chen X Eksioglu EA Zhou J Induction of myelodysplasia by myeloid-derived suppressor cells. *J Clin Invest.* 2013;123:4595–4611. 24216507 10.1172/JCI67580 PMC3809779

[29] Zhao F Hoechst B Duffy A S100A9 a new marker for monocytic human myeloid-derived suppressor cells. *Immunology.* 2012;136:176–183. 22304731 10.1111/j.1365-2567.2012.03566.x PMC3403264

[30] Sato Y Goto Y Narita N Cancer cells expressing toll-like receptors and the tumor microenvironment. *Cancer Microenviron.* 2009;2:S205–S214. 10.1007/s12307-009-0022-y PMC2756339 19685283

[31] Kittang AO Kordasti S Sand KE Expansion of myeloid derived suppressor cells correlates with number of T regulatory cells and disease progression in myelodysplastic syndrome. *Oncoimmunology.* 2016;5:1–9. 10.1080/2162402X.2015.1062208 PMC4801428 27057428

[32] Mei Y Zhao B Basiorka AA Age-related inflammatory bone marrow microenvironment induces ineffective erythropoiesis mimicking del(5q) MDS. *Leukemia.* 2018;32:1023–1033. 29263441 10.1038/leu.2017.326 PMC5886057

[33] Sinha P Okoro C Foell D Proinflammatory S100 proteins regulate the accumulation of myeloid-derived suppressor cells. *J Immunol.* 2008;181:4666–4675. 18802069 10.4049/jimmunol.181.7.4666 PMC2810501

[34] Sand K Theorell J Bruserud Ø Reduced potency of cytotoxic T lymphocytes from patients with high-risk myelodysplastic syndromes. *Cancer Immunol Immunother.* 2016;65:1135–1147. 27481108 10.1007/s00262-016-1865-y PMC11029614

[35] Bontkes HJ Ruben JM Alhan C Azacitidine differentially affects CD4 pos T-cell polarization in vitro and in vivo in high risk myelodysplastic syndromes. *Leuk Res.* 2012;36:921–930. 22503132 10.1016/j.leukres.2012.03.026

[36] Eksioglu EA Chen X Heider KH Novel therapeutic approach to improve hematopoiesis in low risk MDS by targeting MDSCs with the Fc-engineered CD33 antibody B. *Leukemia.* 2017;31:2172–2180. 28096534 10.1038/leu.2017.21 PMC5552472

[37] Movahedi K Guilliams M Bossche J Identification of discrete tumor-induced myeloid-derived suppressor cell subpopulations with distinct T cell-suppressive activity. *Blood.* 2012;111:4233–4244. 10.1182/blood-2007-07-099226 18272812

[38] Youn J-I Nagaraj S Collazo M Subsets of myeloid-derived suppressor cells in tumor-bearing mice. *J Immunol.* 2008;181:5791–5802. 18832739 10.4049/jimmunol.181.8.5791 PMC2575748

[39] Serafini P Mgebroff S Noonan K Myeloid-derived suppressor cells promote cross-tolerance in B-cell lymphoma by expanding regulatory T cells. *Cancer Res.* 2008;68:5439–5449. 18593947 10.1158/0008-5472.CAN-07-6621 PMC2887390

[40] Marini O Spina C Mimiola E Identification of granulocytic myeloid-derived suppressor cells (G-MDSCs) in the peripheral blood of Hodgkin and non-Hodgkin lymphoma patients. *Oncotarget.* 2016;7:27676–27688. 27050283 10.18632/oncotarget.8507 PMC5053680

[41] Azzaoui I Uhel F Rossille D T-cell defect in diffuse large B-cell lymphomas involves expansion of myeloid derived suppressor cells expressing IL-10, PD-L1 and S100A12. *Blood.* 2016;128:1081–1092. 27338100 10.1182/blood-2015-08-662783

[42] Lin Y Gustafson MP Bulur PA Immunosuppressive CD14^+^HLA-DR^low/−^ monocytes in B-cell non-Hodgkin lymphoma. *Blood.* 2011;117:872–881. 21063024 10.1182/blood-2010-05-283820 PMC3035079

[43] Gustafson MP Abraham RS Lin Y Association of an increased frequency of CD14+ HLA-DR^lo/neg^ monocytes with decreased time to progression in chronic lymphocytic leukaemia (CLL). *Br J Haematol.* 2012;156:674–676. 22050346 10.1111/j.1365-2141.2011.08902.x PMC3433277

[44] Jitschin R Braun M Büttner M CLL-cells induce IDOhi CD14+ HLA-DRlo myeloid derived suppressor cells that inhibit T-cell responses and promote TRegs. *Blood.* 2014;124:750–760. 24850760 10.1182/blood-2013-12-546416

[45] Romano A Parrinello NL Vetro C Circulating myeloid-derived suppressor cells correlate with clinical outcome in Hodgkin Lymphoma patients treated up-front with a risk-adapted strategy. *Br J Haematol.* 2015;168:689–700. 25376846 10.1111/bjh.13198

[46] Betsch A Rutgeerts O Fevery S Myeloid-derived suppressor cells in lymphoma: the good, the bad and the ugly. *Blood Rev.* 2018;32:490–498. 29691090 10.1016/j.blre.2018.04.006

[47] Gustafson MP Lin Y Maas ML A method for identification and analysis of non-overlapping myeloid immunophenotypes in humans. *PLoS ONE.* 2015;10:1–19. 10.1371/journal.pone.0121546 PMC4370675 25799053

[48] Tadmor T Fell R Polliack A Absolute monocytosis at diagnosis correlates with survival in diffuse large B-cell lymphoma-possible link with monocytic myeloid-derived suppressor cells. *Hematol Oncol.* 2013;31:325–331. 10.1002/hon.2019 22714941

[49] Wu C Wu X Liu X Prognostic significance of monocytes and monocytic myeloid-derived suppressor cells in diffuse large B-cell lymphoma treated with R-CHOP. *Cell Physiol Biochem.* 2016;39:521–530. 27383764 10.1159/000445644

[50] Geskin LJ Akilov OE Kwon S Therapeutic reduction of cell-mediated immunosuppression in mycosis fungoides and Sézary syndrome. *Cancer Immunol Immunother.* 2018;67:423–434. 29204699 10.1007/s00262-017-2090-z PMC8274400

[51] Raab MS Podar K Breitkreutz I Multiple myeloma. *Lancet.* 2009;374:324–339. 19541364 10.1016/S0140-6736(09)60221-X

[52] Botta C GullÃ A Correale P Myeloid-derived suppressor cells in multiple myeloma: pre-clinical research and translational opportunities. *Front Oncol.* 2014;4:1–12. 25538892 10.3389/fonc.2014.00348 PMC4258997

[53] Yazdani Y Mohammadnia-Afrouzi M Yousefi M Myeloid-derived suppressor cells in B cell malignancies. *Tumor Biol.* 2015;36:7339–7353. 10.1007/s13277-015-4004-z 26330296

[54] Malek E de Lima M Letterio JJ Myeloid-derived suppressor cells: the green light for myeloma immune escape. *Blood Rev.* 2016;30:341–348. 27132116 10.1016/j.blre.2016.04.002 PMC6411302

[55] De Veirman K Van Ginderachter JA Lub S Multiple myeloma induces Mcl-1 expression and survival of myeloid-derived suppressor cells. *Oncotarget.* 2015;6:10532–10547. 25871384 10.18632/oncotarget.3300 PMC4496373

[56] Van Valckenborgh E Schouppe E Movahedi K Multiple myeloma induces the immunosuppressive capacity of distinct myeloid-derived suppressor cell subpopulations in the bone marrow. *Leukemia.* 2012;26:2424–2428. 22522789 10.1038/leu.2012.113

[57] Ramachandran IR Condamine T Lin C Bone marrow PMN-MDSCs and neutrophils are functionally similar in protection of multiple myeloma from chemotherapy. *Cancer Lett.* 2016;371:117–124. 26639197 10.1016/j.canlet.2015.10.040 PMC4919899

[58] Wang J De Veirman K Faict S Multiple myeloma exosomes establish a favourable bone marrow microenvironment with enhanced angiogenesis and immunosuppression. *J Pathol.* 2016;239:162–173. 26956697 10.1002/path.4712

[59] Binsfeld M Muller J Lamour V Granulocytic myeloid-derived suppressor cells angiogenesis in the context of multiple myeloma promote angiogenesis in the context of multiple myeloma. *Oncotarget.* 2016;7:37931–37943. 27177328 10.18632/oncotarget.9270 PMC5122361

[60] Wang J De Veirman K De Beule N The bone marrow microenvironment enhances multiple myeloma progression by exosome-mediated activation of myeloid-derived suppressor cells. *Oncotarget.* 2015;6:43992–44004. 26556857 10.18632/oncotarget.6083 PMC4791281

[61] Xu Y Zhang X Liu H Mesenchymal stromal cells enhance the suppressive effects of myeloid-derived suppressor cells of multiple myeloma. *Leuk Lymphoma.* 2017;58:2668–2676. 28317413 10.1080/10428194.2017.1298753

[62] Zhuang J Zhang J Lwin ST Osteoclasts in multiple myeloma are derived from Gr-1+CD11b+ myeloid-derived suppressor cells. *PLoS ONE.* 2012;7:e48871. 23173040 10.1371/journal.pone.0048871 PMC3500251

[63] Ramachandran I Martner A Pisklakova A Myeloid derived suppressor cells regulate growth of multiple myeloma by inhibiting T cells in bone marrow. *J Immunol.* 2013;190:3815–3823. 23460744 10.4049/jimmunol.1203373 PMC3608837

[64] Serafini P Meckel K Kelso M Phosphodiesterase-5 inhibition augments endogenous antitumor immunity by reducing myeloid-derived suppressor cell function. *J Exp Med.* 2006;203:2691–2702. 17101732 10.1084/jem.20061104 PMC2118163

[65] Giallongo C Tibullo D Parrinello NL Granulocyte-like myeloid derived suppressor cells (G-MDSC) are increased in multiple myeloma and are driven by dysfunctional mesenchymal stem cells (MSC). *Oncotarget.* 2016;7:85764–85775. 26967390 10.18632/oncotarget.7969 PMC5349872

[66] G̈or̈gun GT Whitehill G Anderson JL Tumor-promoting immune-suppressive myeloid-derived suppressor cells in the multiple myeloma microenvironment in humans. *Blood.* 2013;121:2975–2987. 23321256 10.1182/blood-2012-08-448548 PMC3624943

[67] Favaloro J Liyadipitiya T Brown R Myeloid derived suppressor cells are numerically, functionally and phenotypically different in patients with multiple myeloma. *Leuk Lymphoma.* 2014;55:2893–2900. 24625328 10.3109/10428194.2014.904511

[68] Wang Z Zhang L Wang H Tumor-induced CD14+HLA-DR−/low myeloid-derived suppressor cells correlate with tumor progression and outcome of therapy in multiple myeloma patients. *Cancer Immunol Immunother.* 2015;64:389–399. 25548095 10.1007/s00262-014-1646-4 PMC11028624

[69] Karpatkin S Autoimmune (idiopathic) thrombocytopenic purpura. *Lancet.* 1997;349:1531–1536. 9167472 10.1016/S0140-6736(96)12118-8

[70] Hou Y Feng Q Xu M High-dose dexamethasone corrects impaired myeloid-derived suppressor cell function via Ets1 in immune thrombocytopenia. *Blood.* 2016;127:1587–1597. 26744458 10.1182/blood-2015-10-674531

[71] Zhou J Zhou Y Wen J Circulating myeloid-derived suppressor cells predict disease activity and treatment response in patients with immune thrombocytopenia. *Brazilian J Med Biol Res.* 2017;50:2–7. 10.1590/1414-431X20165637 PMC5343560 28225866

[72] Shao X Wu B Cheng L Distinct alterations of CD68+CD163+ M2-like macrophages and myeloid-derived suppressor cells in newly diagnosed primary immune thrombocytopenia with or without CR after high-dose dexamethasone treatment. *J Transl Med.* 2018;16:1–11. 29499727 10.1186/s12967-018-1424-8 PMC5833082

[73] Aslam R Burack WR Segel GB Intravenous immunoglobulin treatment of spleen cells from patients with immune thrombocytopenia significantly increases the percentage of myeloid-derived suppressor cells. *Br J Haematol.* 2018;181:262–264. 28165136 10.1111/bjh.14542

[74] Papadaki HA Stamatopoulos K Damianaki A Activated T-lymphocytes with myelosuppressive properties in patients with chronic idiopathic neutropenia. *Br J Haematol.* 2005;128:863–876. 15755293 10.1111/j.1365-2141.2005.05380.x

[75] Bizymi N Velegraki M Damianaki A Low proportion of myeloid derived suppressor cell populations in the peripheral blood of patients with chronic idiopathic neutropenia. *HemaSphere.* 2018;2 suppl 1:103–104.

[76] Xin J Breslin P Wei W Necroptosis in spontaneously-mutated hematopoietic cells induces autoimmune bone marrow failure in mice. *Haematologica.* 2017;102:295–307. 27634200 10.3324/haematol.2016.151514 PMC5286937

[77] Ferrara JLM Levine JE Reddy P Graft-versus-host disease. *Lancet.* 2009;373:1550–1561. 19282026 10.1016/S0140-6736(09)60237-3 PMC2735047

[78] Koehn BH Blazar BR Role of myeloid-derived suppressor cells in allogeneic hematopoietic cell transplantation. *J Leukoc Biol.* 2017;102:335–341. 28148718 10.1189/jlb.5MR1116-464R PMC5505741

[79] Blazar BR MacDonald KPA Hill GR Immune regulatory cell infusion for graft-versus-host disease prevention and therapy. *Blood.* 2018;131:2651–2660. 29728401 10.1182/blood-2017-11-785865 PMC6032895

[80] Luyckx A Schouppe E Rutgeerts O G-CSF stem cell mobilization in human donors induces polymorphonuclear and mononuclear myeloid-derived suppressor cells. *Clin Immunol.* 2012;143:83–87. 22341087 10.1016/j.clim.2012.01.011

[81] Vendramin A Gimondi S Bermema A Graft monocytic myeloid-derived suppressor cell content predicts the risk of acute graft-versus-host disease after allogeneic transplantation of granulocyte colony-stimulating factor-mobilized peripheral blood stem cells. *Biol Blood Marrow Transplant.* 2014;20:2049–2055. 25246295 10.1016/j.bbmt.2014.09.011

[82] Lv M Zhao X-S Hu Y Monocytic and promyelocytic myeloid-derived suppressor cells may contribute to G-CSF-induced immune tolerance in haplo-identical allogeneic hematopoietic stem cell transplantation. *Am J Hematol.* 2015;90:E9–E16. 25303038 10.1002/ajh.23865

[83] Yin J Wang C Huang M Circulating CD14(+) HLA-DR(−/low) myeloid-derived suppressor cells in leukemia patients with allogeneic hematopoietic stem cell transplantation: novel clinical potential strategies for the prevention and cellular therapy of graft-versus-host disease. *Cancer Med.* 2016;5:1654–1669. 27109254 10.1002/cam4.688 PMC4944894

[84] Highfill SL Rodriguez PC Zhou Q Bone marrow myeloid-derived suppressor cells (MDSCs) inhibit graft-versus-host disease (GVHD) via an arginase-1-dependent mechanism that is up-regulated by interleukin-13. *Blood.* 2016;116:5738–5748. 10.1182/blood-2010-06-287839 PMC3031417 20807889

[85] Wang D Yu Y Haarberg K Dynamic change and impact of myeloid-derived suppressor cells in allogeneic bone marrow transplantation in mice. *Biol Blood Marrow Transplant.* 2013;19:692–702. 23376089 10.1016/j.bbmt.2013.01.008 PMC4011929

[86] Le Blanc K Jitschin R Mougiakakos D Myeloid-derived suppressor cells in allogeneic hematopoietic stem cell transplantation: a double-edged sword? *Oncoimmunology.* 2013;2:7–9. 10.4161/onci.25009 PMC3782166 24073377

[87] Kusmartsev S Cheng F Yu B All-trans-retinoic acid eliminates immature myeloid cells from tumor-bearing mice and improves the effect of vaccination. *Cancer Res.* 2003;63:4441–4449. 12907617

[88] Nefedova Y Fishman M Sherman S Mechanism of all-trans retinoic acid effect on tumor-associated myeloid-derived suppressor cells. *Cancer Res.* 2007;67:11021–11028. 18006848 10.1158/0008-5472.CAN-07-2593

[89] Lee J-M Seo J-H Kim Y-J The restoration of myeloid-derived suppressor cells as functional antigen-presenting cells by NKT cell help and all-trans-retinoic acid treatment. *Int J Cancer.* 2012;131:741–751. 21898392 10.1002/ijc.26411

[90] Iclozan C Antonia S Chiappori A Therapeutic regulation of myeloid-derived suppressor cells and immune response to cancer vaccine in patients with extensive stage small cell lung cancer. *Cancer Immunol Immunother.* 2009;6:247–253. 10.1007/s00262-013-1396-8 PMC3662237 23589106

[91] Tomihara K Fuse H Heshiki W Gemcitabine chemotherapy induces phenotypic alterations of tumor cells that facilitate antitumor T cell responses in a mouse model of oral cancer. *Oral Oncol.* 2014;50:457–467. 24582211 10.1016/j.oraloncology.2014.01.013

[92] Annels NE Shaw VE Gabitass RF The effects of gemcitabine and capecitabine combination chemotherapy and of low-dose adjuvant GM-CSF on the levels of myeloid-derived suppressor cells in patients with advanced pancreatic cancer. *Cancer Immunol Immunother.* 2014;63:175–183. 24292263 10.1007/s00262-013-1502-y PMC11028876

[93] Porembka MR Mitchem JB Belt BA Pancreatic adenocarcinoma induces bone marrow mobilization of myeloid derived suppressor cells which promote primary tumor growth. *Cancer Immunol Immunother.* 2012;61:1373–1385. 22215137 10.1007/s00262-011-1178-0 PMC3697836

[94] Krejcik J Casneuf T Nijhof IS Daratumumab depletes CD38^+^ immune-regulatory cells, promotes T-cell expansion, and skews T-cell repertoire in multiple myeloma. *Blood.* 2016;128:384–395. 27222480 10.1182/blood-2015-12-687749 PMC4957162

[95] Trikha P Carson WE Signaling pathways involved in MDSC regulation. *Biochim Biophys Acta.* 2014;1846:55–65. 24727385 10.1016/j.bbcan.2014.04.003 PMC4140957

[96] Youn J Kumar V Collazo M Epigenetic silencing of retinoblastoma gene regulates pathologic differentiation of myeloid cells in cancer. *Nat immunol.* 2013;14:211–220. 23354483 10.1038/ni.2526 PMC3578019

[97] Stone RM Manley PW Larson RA Midostaurin: its odyssey from discovery to approval for treating acute myeloid leukemia and advanced systemic mastocytosis. *Blood Adv.* 2018;2:444–453. 29487059 10.1182/bloodadvances.2017011080 PMC5858474

[98] Rosborough BR Mathews LR Matta BM FLT3 ligand mediates STAT3-independent expansion, but STAT-3 dependent activation of myeloid-derived suppressor cells. *J Immunol.* 2014;192:3470–3473. 24639346 10.4049/jimmunol.1300058 PMC3994403

[99] Duffy A Zhao F Haile L Comparative analysis of monocytic and granulocytic myeloid-derived suppressor cell subsets in patients with gastrointestinal malignancies. *Cancer Immunol Immunother.* 2013;62:299–307. 23011590 10.1007/s00262-012-1332-3 PMC6628699

[100] Bruger AM Dorhoi A Esendagli G How to measure the immunosuppressive activity of MDSC: assays, problems and potential solutions. *Cancer Immunol Immunother*. 2018; 10.1007/s00262-018-2170-8. PMC11028070 29785656

